# When Cigarette Smoking Meets COVID-19: How the Two Types of Threat and Efficacy Perceptions Interactively Predict Danger Control and Fear Control Processes

**DOI:** 10.3390/ijerph20042970

**Published:** 2023-02-08

**Authors:** Yachao Li, Hue Trong Duong, Zachary B. Massey, Victoria Churchill, Lucy Popova

**Affiliations:** 1 Department of Communication Studies, Department of Public Health, The College of New Jersey, Ewing Township, NJ 08628, USA; 2 Department of Communication, Georgia State University, Atlanta, GA 30302, USA; 3 School of Journalism, University of Missouri, Columbia, MO 65201, USA; 4 Cancer Health Equity Institute, Morehouse School of Medicine, Atlanta, GA 30310, USA; 5 School of Public Health, Georgia State University, Atlanta, GA 30302, USA

**Keywords:** COVID-19, smoking, smoking cessation, EPPM, danger control, fear control

## Abstract

Growing evidence indicates that communicating the combined risk of smoking and COVID-19 encourages smoking cessation. Guided by the Extended Parallel Process Model (EPPM), we examined how perceived threats of smoking and COVID-19 independently and interactively predicted danger control responses (i.e., quit intentions and COVID-19-protective behavioral intentions) and fear control responses (i.e., fear and fatalism). We also explored the direct and interactive impacts of perceived efficacy of quitting smoking and COVID-protective behaviors on message outcomes. Structural equation modeling results (*N* = 747 U.S. adults who smoke) indicated that the perceived efficacy of COVID-protective behaviors positively predicted quit intentions. Higher perceived threat of COVID-19 and greater quitting efficacy predicted higher quit intentions directly and indirectly via fear. As perceived COVID-protective efficacy increased, the positive association between perceived quitting efficacy and quit intentions also increased. Smoking-related threat and efficacy perceptions did not predict COVID-protective behavioral intentions. This study added to EPPM by considering how threat and efficacy perceptions deriving from two different yet closely related risks affect protective behaviors. Thus, combining multiple threats in a single message might be a promising strategy to motivate smoking cessation amid the pandemic.

## 1. Introduction

The coronavirus disease 2019 (COVID-19) continues to adversely affect public health and well-being. A growing body of evidence shows that smoking cigarettes worsens the severity of COVID-19 [1,2,3,4]. However, people’s awareness of this risk varies widely, ranging from being highly cognizant to having never heard of it [5,6]. Because awareness about the increased COVID-19 severity for people who smoke is positively related to intentions to quit smoking [7,8,9], communicating the risk of smoking for worsening COVID-19 to people who smoke might be a pathway to motivate smoking cessation.

Emerging research has examined how messaging on the combined risks of smoking and COVID-19 influenced smoking-related intentions. For instance, among people who smoke and/or vape, exposure to messages with cigarette smoking risk or COVID-19 risk elicited greater perceived message effectiveness for discouraging smoking than generic tobacco-product messages (control) [10]. Researchers also found that compared with smoking cessation messages that referred to the risk of chest infection and financial cost, exposure to COVID-19-related messages increased intentions to quit smoking [11]. Warning messages on the harm of COVID-19 to those who vape were rated higher on perceived message effectiveness than nicotine addiction messages [12]. Similarly, exposure to messages communicating the risk of smoking for COVID-19 severity resulted in higher intentions to quit smoking versus COVID-19 risk messages alone and greater intentions to reduce cigarette use than exclusive smoking risk messages [13]. Thus, there is evidence that warning about the risk of COVID-19 severity from smoking can impact intentions among people who smoke. However, no published research has analyzed theoretical mechanisms to explain these message effects.

The Extended Parallel Process Model (EPPM) [14] is useful for investigating effects of health messages that evoke strong emotions. Specifically, the EPPM argues that perceived threat and efficacy are two underlying mechanisms accounting for whether people accept or reject health recommendations in messages that evoke fear. If perceived threat is high but perceived efficacy is low, people will reject the message to ease their fear (i.e., fear control). If the perceived threat is high and perceived efficacy are high, people will follow message recommendations to alleviate risk from the threat (i.e., danger control) [14]. Existing EPPM research typically tests how perceived threat of a health problem (e.g., perceived threat of lung cancer) and efficacy of a suggested behavior (e.g., perceived efficacy of quitting smoking) affect people’s attitudes, intentions, and behaviors (e.g., smoking cessation). Predominantly, prior studies have focused on the effects of a single threat.

However, in the COVID-19 pandemic, people who smoke are susceptible to two threats—the threat of smoking and the threat of COVID-19. For them, these two threats are related because smoking exacerbates the severity of COVID-19. Accordingly, the EPPM would suggest that people who smoke should process threat and efficacy information about two related threats—smoking-related diseases *and* worse COVID-19 severity from smoking cigarettes. Nevertheless, few studies have considered how threat and efficacy perceptions deriving from two different yet closely related risks affect attitudes and protective behaviors.

Thus, this study aims to examine how the perceived threat of smoking and COVID-19 independently and interactively predicted danger control and fear control responses. Another research goal is to explore the direct and interactive impacts of perceived efficacy of quitting smoking and COVID-protective behaviors on message outcomes. To achieve our research objectives, we utilized structural equation modeling (SEM) to test the EPPM as an omnibus model, simultaneously assessing the direct and joint impacts of different threat and efficacy perceptions on two health behavioral intentions. The results add to research on EPPM and provide insights into how combined risk messages may effectively reduce smoking and promote COVID-19-protective behaviors.

### 1.1. Danger Control Responses

Within the EPPM framework, perceived threat is a cognitive appraisal of the danger within a health message. Perceived threat consists of perceived susceptibility and severity of the threat. Perceived susceptibility is defined as beliefs about the probability of personally experiencing a threat. Perceived severity refers to the perceived gravity of the threat’s adverse consequences. When people believe a health issue presents a personally relevant, serious problem (i.e., high perceived threat), they experience fear and assess the likelihood and abilities to mitigate or avoid the threat (i.e., perceived efficacy). Perceived efficacy comprises two dimensions: perceived response efficacy (i.e., the beliefs about how effective an action is in alleviating a threat) and perceived self-efficacy (i.e., the perceived ability to carry out the recommended behavior). The EPPM argues that when perceived threat is high and perceived efficacy is also high, people engage in danger control by developing positive attitudes and adopting advocated protective behaviors to avert the danger [14].

Meta-analyses showed that perceived threat and efficacy are positively related to behavioral intentions [15,16,17]. In the smoking context, higher perceived threat of smoking-related disease [18,19,20] and higher perceived efficacy of quitting [21,22] predict greater intentions to quit. Similarly, in the context of COVID-19, as the perceived threat of COVID-19 and perceived efficacy of social distancing increased, people reported more intentions to socially distance [23]. Perceived severity of COVID-19 was also related to higher readiness to quit smoking and increased quit attempts [24]. Given that smoking cigarettes worsens the severity of COVID-19, concerns about the adverse consequences of COVID-19 may motivate people who smoke to quit. As people simultaneously consider threats of smoking and COVID-19 while believing that they can effectively protect themselves from those threats by not smoking and practicing protective measures, they may be more likely to quit smoking and engage in COVID-19-protective behaviors. The two perceived threats of smoking and COVID-19 might operate in an interactive way; for example, when the perceived threat of smoking is low, adding high perceived threat of COVID-19 might increase quitting intentions slightly; however, when the perceived threat of smoking is high, combining it with a high perceived threat of COVID-19 could greatly increase quitting intentions. As such, the two perceived threats can influence each other, resulting in a multiplicative effect. Similarly, the two efficacy perceptions might feed off of each other and result in even greater positive outcomes than the simple additive effect. Thus, we predict:

**H1.**  *Perceived threat of smoking (a), perceived threat of COVID-19 (b), the interaction between the two threat perceptions (c), perceived efficacy of quitting smoking (d), perceived efficacy of COVID-protective behaviors (e), and the interaction between the two efficacy perceptions (f) positively predict intentions to quit smoking*.

**H2.**  *Perceived threat of smoking (a), perceived threat of COVID-19 (b), the interaction between the two threat perceptions (c), perceived efficacy of quitting smoking (d), perceived efficacy of COVID-protective behaviors (e), and the interaction between the two efficacy perceptions (f) positively predict intentions to engage in COVID-protective behaviors (i.e., wearing face masks, washing hands frequently, and social distancing).*

### 1.2. Fear Control Responses

According to the EPPM, when people perceive high threat of a disease but low efficacy of a recommended behavior, they are more likely to engage in fear control responses through maladaptive coping strategies to reduce fear [14]. In other words, when people believe there are no efficacious methods to eliminate the danger, they engage in a defensive mechanism to control fear instead of taking protective actions to alleviate the threat [14]. Prior meta-analysis has indicated that perceived threat is positively related to fear [15]. Thus, when people who smoke are faced with both smoking and COVID-19 threats, it is likely that they will feel more fearful when they perceive both threats as high.

**H3.**  *Perceived threat of smoking (a), perceived threat of COVID-19 (b), and the interaction between the two threat perceptions (c) positively predict fear.*

The EPPM also posits that when perceived efficacy (a combination of both self-efficacy and response efficacy) is high, people are less fearful of a health risk. This proposition has rarely been tested, with only a few studies providing inconsistent results. For instance, Rippetoe and Rogers revealed that self-efficacy and response efficacy are negatively related to fear [25]. In contrast, researchers found that response efficacy, but not self-efficacy, positively predicted fear [26]. In an experimental study, perceived efficacy was positively correlated with fear of health consequences of smokeless tobacco, but not related to fear during exposure to anti-tobacco messages [27]. However, other studies using SEM to test the EPPM as an omnibus model found no relationship between perceived efficacy and fear [23,28]. Thus, we ask:

**RQ1.** How do perceived efficacy of quitting smoking (a), perceived efficacy of COVID-protective behaviors (b), and the interaction between the two efficacy perceptions (c) predict fear?

The literature on EPPM suggests that fatalism is one major maladaptive coping reaction when people are exposed to overwhelming threats. Fatalism refers to “the acceptance of a stressful situation as unchangeable and complacency in the face of danger because nothing can be done anyway” (p. 598) [25]. That is, individuals with fatalistic beliefs tend to accept adverse outcomes and will not likely engage in behaviors aimed at reducing the threat. This is in line with EPPM’s proposition that “fear causes maladaptive responses” (p. 343) [14]. Empirical work, however, found inconclusive evidence for this proposition. For instance, while some researchers observed a positive association between fear and message derogation (one type of maladaptive response) [29], other studies reported negative relationships between fear and defensive avoidance [23,26]. The inconsistencies have been raised in Popova’s review of 29 EPPM studies [30]. Of the seven studies testing the prediction that fear would lead to maladaptive responses, two contradicted, three found mixed support for, and two supported the proposition. Thus, we ask:

**RQ2.** How is fear associated with fatalism?

**Figure 1:**
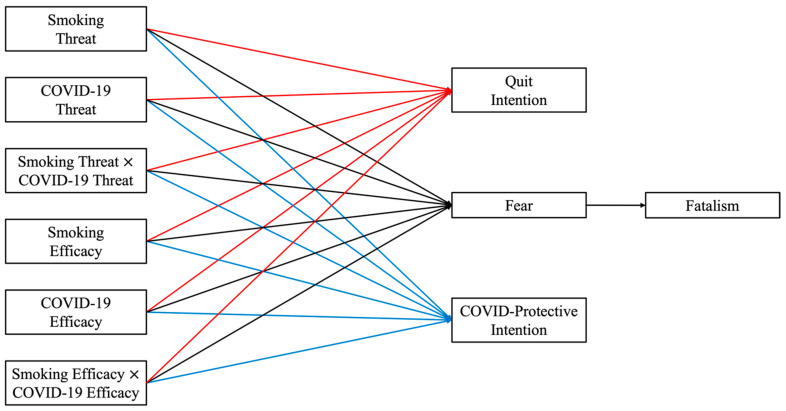
Conceptual model. Note: Paths are colored for visual clarity. Smoking threat = perceived threat of smoking, COVID-19 threat = perceived threat of COVID-19, smoking threat × COVID-19 threat = the interaction between perceived threat of smoking and COVID-19, smoking efficacy = perceived efficacy of quitting smoking, COVID-19 efficacy = perceived efficacy of COVID-19-protective behaviors, smoking efficacy × COVID-19 efficacy = the interaction between the perceived efficacy of quitting smoking and COVID-19-protective behaviors.

Figure 1 summarizes our conceptual model.

## 2. Materials and Methods

### 2.1. Partippants

This study was part of a larger data collection investigating how people who smoke respond to news stories about increased COVID-19 severity from smoking [13,31,32]. The primary study had three risk conditions showing messages about the risk of COVID-19, the risk of smoking, or the combined risk (i.e., smoking exacerbates COVID-19 serious outcomes), and a control condition with non-risk messages (e.g., facts about architecture). This study analyzed data in the risk message conditions (*N* = 747) and excluded data from the control condition (*n* = 257).

The sample comprised U.S. adults who currently smoke. Participants were recruited by the marketing company Toluna (www.toluna-group.com, accessed on 14 January 2023) in August 2020. Toluna uses multiple strategies (e.g., web banners, website referrals, affiliate marketing) to recruit eligible participants. Inclusion criteria for the study were being 18 years old or older, having smoked 100 cigarettes in their lifetime and currently smoking cigarettes every day or some days, and being able and willing to participate in a study conducted in English. To recruit participants, the quota sampling method was used based on gender, age, education, race, and income categories that approximated the distributions in the national population.

### 2.2. Ethics

All research team members completed the Human Subject Projection training and obtained the certificate before the study started. The study protocol was approved by the Institutional Review Board (IRB) of Georgia State University (Approval # H19055). All participants completed an electronic informed consent.

### 2.3. Design and Procedures

We tested the questionnaire for feasibility and readability in a pilot study (*n* = 100). The results showed that the study was feasible to conduct in under 20 min (median time of the pilot was 14 min) and that the questions were understandable and easy to answer (we added an explanation to one question). The primary experiment was hosted online through the Toluna system. Participants provided consent, completed demographic measures, and answered pretest measures of smoking frequency, smoking quit intentions, previously having COVID-19, fear of COVID-19, political ideology, self-reported health status, and psychological distress. Participants were then randomized into one of four conditions: (1) COVID-19 risk, (2) smoking risk, (3) combined risk (i.e., smoking increases COVID-19 severity), and (4) control. Each condition had five messages to avoid case-category confounding issues, [33] with participants randomly exposed to one of the five messages in each condition.

Following message exposure, participants completed posttest measures of perceived threat, perceived efficacy, fear, fatalism, intentions to quit smoking, and intentions to engage in COVID-19-protective behaviors. Participants were debriefed at the end of the questionnaire and informed that the messages were for research purposes only and not approved by any public health or federal agency. The debrief included information about the increased severity of COVID-19 for people who smoke, along with a smoking quit line number and links to smoking cessation websites.

### 2.4. Message Stimuli

The experimental stimuli (e.g., messages) were adapted from news stories (e.g., *ABC*, *New York Times*, and *Fox News*) and educational campaigns about smoking risks [34]. The messages were configured as online news stories, approximately 150–250 words in length, with headlines and color photos. There was no reference to any specific journalists or news agencies to avoid confounding effects of message credibility. Messages in the smoking risk condition explained the negative health impacts of smoking (i.e., lung and heart disease, cancer, and death). Messages about the COVID-19 risk condition described the disease progression (i.e., lung failure, heart damage, and death). The combined risk condition described how smoking makes COVID-19 worse. The control condition showed non-risk messages (e.g., facts about architecture) and is not included in this paper. Each risk message concluded with an efficacy paragraph emphasizing useful ways to deal with the described risks, including quitting smoking, taking protective precautions against COVID-19, or quitting smoking and protecting against COVID-19 infection. Study messages are reported elsewhere [13].

### 2.5. Measures

Table 1 reports variable means and standardized deviations. Unless stated otherwise, variables were measured on a scale of 1 (*not at all*) to 9 (*extremely*).

#### 2.5.1. Perceived Threat of Smoking

Two items were averaged to assess perceived threat of smoking: perceived severity (i.e., “If you develop a smoking-related disease, how severe or serious will it be?”) and perceived susceptibility (i.e., “How likely is it for you to develop a smoking-related disease?”) (*r* = 0.57. *p* < 0.001) [30].

#### 2.5.2. Perceived Threat of COVID-19

Two items were averaged to measure the perceived threat of COVID-19: perceived severity (i.e., “If you catch COVID-19 [coronavirus], how severe or serious will it be?”) and perceived susceptibility (i.e., “How likely is it for you to catch COVID-19 [coronavirus]?”) (*r* = 0.38. *p* < 0.001) [22].

#### 2.5.3. Perceived Efficacy of Quitting Smoking

Two items assessed the self-efficacy of quitting smoking (e.g., “It is easy for me to quit smoking”; *r* = 0.50. *p* < 0.001; *M* = 4.86, *SD* = 2.23). Two items measured response efficacy (e.g., “Quitting smoking is effective in preventing cancer;” *r* = 0.52. *p* < 0.001; *M* = 6.94, *SD* = 1.93). Self-efficacy and response efficacy scores were averaged, with higher numbers indicating a higher perceived efficacy of quitting.

#### 2.5.4. Perceived Efficacy of COVID-19-Protective Behaviors

To assess self-efficacy of COVID-protective behaviors, participants indicated their confidence to “wear a face mask in public”, “wash my hands frequently”, and “practice social distancing” (α = 0.85; *M* = 7.29, *SD* = 1.56). Respondents also rated the effectiveness of these three measures to prevent COVID-19 infection to index response efficacy (α = 0.84; *M* = 7.41, *SD* = 1.78). Self-efficacy and response efficacy scores were averaged, with higher numbers meaning higher perceived efficacy of COVID-19-protective behaviors.

#### 2.5.5. Fear

Fear was measured using one item asking participants how they felt while looking at the news story [35].

#### 2.5.6. Fatalism

A 7-point scale (1 = *strongly disagree*, 7 = *strongly agree*) assessed fatalism. Items included, “If someone is meant to get a serious disease, they will get it no matter what they do”, “How long I live is a matter of luck”, and “Everything that can go wrong for me does” (α = 0.69) [36].

#### 2.5.7. Quit Intentions

After message exposure, participants answered how likely in the next six months they would: “Reduce the number of cigarettes you smoke in a day”; “Use nicotine gum, nicotine patch, or other forms of nicotine replacement therapy”; and “Seek counseling/support to help you quit smoking” (α = 0.75) [21].

#### 2.5.8. COVID-Protective Intentions

Participants were given the following prompt: “How frequently do you intend to do each of the following in the next two weeks if the COVID-19 pandemic continues?” On a 4-point scale (1 = *Never*, 4 = *Always*), participants responded to three items: “Wear a face mask in public”, “Wash hands with soap and water”, and “Practice social distancing” (α = 0.73).

#### 2.5.9. Covariates

Covariates were measured at pretest and included demographic measures of biological sex (0 = *male*, 1 = *female*), age (continuous), race (0 = *non-Hispanic White*, 1 = *People of color*), and education (0 = *high school or less*, 1 = *greater than high school*). We measured additional variables that could be predictive of outcomes: smoking frequency (0 = *somedays*, 1 = *everyday*), pretest smoking quit intentions (1 = *never expect to quit*, 5 = *currently trying to quit*), COVID-19 status (0 = *never had*, 1 = *had or suspected to have had*), political ideology (1 = *very conservative*, 5 = *very liberal*), health status (1 = *very poor*, 5 = *very good*), fear of COVID-19 (1 = *not at all*, 9 = *extremely*), and serious psychological distress (SPD) [37] (0 = *no SPD*, 1 = *have SPD*). Message conditions, including COVID-19 risk (1 = *COVID-19 risk condition*, 0 = *other conditions*) and smoking risk (1 = *smoking risk*, 0 = *other conditions*), were also controlled for.

## 3. Results

Table 1 presents descriptive statistics and correlations among variables. Following Irwin and McClelland’s [38] recommendation, we mean centered all study variables before computing the interaction term between perceived threat of smoking and perceived threat of COVID-19 and the interaction term between perceived efficacy of quitting and perceived efficacy of COVID-protective behaviors. Interaction terms were computed by multiplying the two variables. Path analyses via Mplus tested the hypotheses and explored the research questions. Data were estimated using maximum likelihood estimation. We used the following criteria for the model fit evaluation: a model with RMSEA ≤ 0.06, CFI ≥ 0.95, TFI ≥ 0.95, and SRMR ≤ 0.05 is considered well-fitted [39].

**Figure 2:**
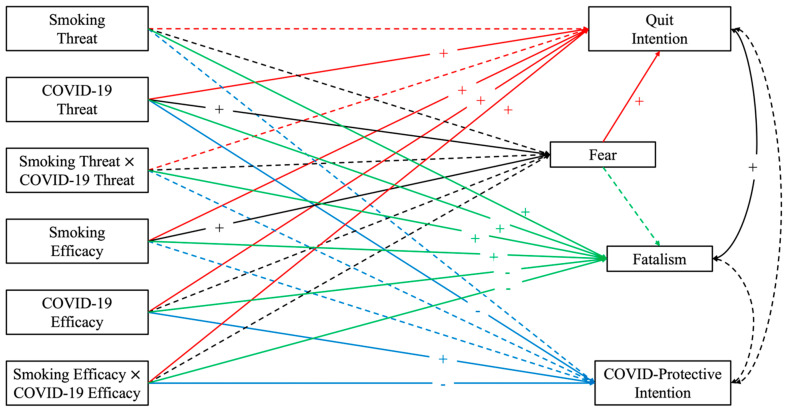
Path analysis results: Significant paths and directions. Note: For visual clarity, certain paths are colored. Solid lines indicate significant paths. Dashed lines are non-significant paths. “+” indicates positive relationships. “-” indicates negative relationships. Smoking threat = perceived threat of smoking, COVID-19 threat = perceived threat of COVID-19, smoking threat × COVID-19 threat = the interaction between perceived threat of smoking and COVID-19, smoking efficacy = perceived efficacy of quitting smoking, COVID-19 efficacy = perceived efficacy of COVID-protective behaviors, smoking efficacy × COVID-19 efficacy = the interaction between the perceived efficacy of quitting smoking and COVID-protective behaviors.

Controlling for covariates, the original model did not fit the data well, χ^2^(8) = 69.073, *p* < 0.001; RMSEA = 0.101, 95% CI [0.080, 0.124]; CFI = 0.951, TLI = 0.496; SRMR = 0.027. Several paths were then added into the model based on the modification indices [40]. The revised model fit the data well, χ^2^(1) = 1.462, *p* = 0.227; RMSEA = 0.025, 95% CI [0.000, 0.104]; CFI = 0.998, TLI = 0.969; SRMR = 0.003. Figure 2 illustrates the significant paths and their direction.

As shown in Table 2, the perceived threat of COVID-19, the perceived efficacy of quitting smoking, the perceived efficacy of COVID-19-protective behaviors, the interaction between the two efficacy perceptions, and fear positively predicted intentions to quit smoking. Thus, H1b, d–f were supported. H1a and H1c were not supported. This significant interaction indicated that as the perceived efficacy of COVID-19-protective behaviors increased, the positive association between the perceived efficacy of quitting and quit intentions also increased.

In addition, perceived threat of smoking, the interaction between the two threat perceptions, and perceived efficacy of quitting did not predict COVID-protective behaviors. H2a, c, and d were not supported. In contrast to H2b, the perceived threat of COVID-19 negatively predicted intentions to engage in COVID-19-protective behaviors. While perceived efficacy of COVID-19-protective behaviors positively predicted behavioral intentions, the interaction between the two efficacy perceptions negatively predicted behavioral intentions. Thus, H2e, but not H2f, was supported. The significant interaction indicated that perceived efficacy of quitting hindered the positive effect of perceived efficacy of COVID-19-protective behaviors on intentions.

There was a positive association between perceived threat of COVID-19 and fear, supporting H3b. However, neither the perceived threat of smoking nor the interaction between the two threat perceptions predicted fear. H3a and c were not supported. RQ1 asked how efficacy perceptions predicted fear. The results showed that perceived efficacy of smoking positively predicted fear. However, there were no relationships between fear and perceived efficacy of COVID-19-protective behaviors, and the efficacy interaction. Post hoc indirect analyses found that fear served as a mechanism through which COVID-19 threat (Effect = 0.019, SE = 0.007, 95% CI: [0.005, 0.033]) and smoking efficacy (Effect = 0.016, SE = 0.006, 95% CI: [0.004, 0.029]) indirectly predicted quit intentions.

RQ2 assessed the relationship between fear and fatalism. No significant relationship between the two was observed. Additionally, perceived threat of smoking, perceived threat of COVID-19, and the threat interaction positively predicted fatalism. That is, one threat perception amplified the positive effects of the other threat perception on fatalism. Moreover, there was a positive relationship between perceived efficacy of quitting and fatalism. The perceived efficacy of COVID-19-protective behaviors and the efficacy interaction negatively predicted fatalism. The significant interaction showed that as perceived efficacy of quitting increased, the strength of the negative association between COVID-19-protective efficacy and fatalism also increased.

## 4. Discussion

This study investigated the effects of fear appeal messages that combine two different but related health issues: smoking and COVID-19. Smoking has been found to increase COVID-19 severity, and agencies like the CDC and WHO have begun messaging on this link to motivate smoking cessation [41]. Using structural equation modeling to assess the effects of perceptions of threat and efficacy related to smoking and COVID-19 on behavioral intentions related to these health issues, this study found some support for the hypotheses based on the propositions of the EPPM but also some contrary findings.

### 4.1. Predicting Quit Intentions

Quit intentions were positively associated with the perceived threat of COVID-19, smoking efficacy, COVID-19 efficacy, and the interaction between smoking and COVID-19 efficacy. The results are consistent with the EPPM’s theorizing that greater threat and efficacy promote danger control responses where attitudes and behaviors align with the message recommendation. Moreover, these findings support messages about COVID-19 severity to motivate smoking cessation among people who smoke, as shown in other studies [10,11,13]. In addition, fear served as a mechanism through which COVID-19 threat and smoking efficacy predicted quit intentions. This is in line with the findings that fear serves as a primary mechanism accounting for the effects of messages communicating threats (such as warning labels on cigarette packs) on behavior-related outcomes [42].

While the interaction effect between two threats on quit intentions was not significant, the interaction effect of smoking and COVID-19 efficacy was. As COVID-19 efficacy increased, the positive relationship between smoking efficacy and quit intention got stronger, indicating a multiplicative effect. During social crises like the COVID-19 pandemic, people often feel overwhelmed and stressed. Thus, when facing multiple threats, people who smoke may continue smoking to relieve this stress [43]. In other words, smoking may be a maladaptive coping mechanism to overcome combined threats. However, if they perceive another effective solution to the additional threat (i.e., high COVID-19 response and self-efficacy), the level of stress might be lower, or they might believe that they can mitigate the threat through an effective response (i.e., social distancing), engaging an adaptive coping mechanism. As a result, high COVID-19 efficacy boosts the effects of smoking efficacy in motivating quitting intentions. Because these two positive thoughts (preventing COVID-19 complications and negative health outcomes from smoking) were likely associated with one another, it was possible that participants experienced a spreading-activation effect in which one cognition triggers another and jointly produced effects on behavioral intentions [44,45]. This result comports with recent research showing that among a sample of U.S. smokers, greater perceptions about smoking-related COVID-19 severity were associated with greater likelihood to smoke, but also greater readiness to quit smoking [24].

### 4.2. Predicting COVID-Protective Intentions

Consistent with the EPPM predictions and previous research [15], COVID-protective intention was positively related to COVID-19 efficacy. While the perceived efficacy of smoking did not directly predict COVID-protective intention, it negatively moderated the positive association between COVID-19 efficacy and intentions to take protective behaviors against COVID-19. In other words, as the efficacy of quitting smoking increased, the positive relationship between COVID-19 efficacy and COVID-protective intention decreased. As messages in the combined risk condition explicitly stated that quitting smoking would reduce the severity of COVID-19 infection, we speculated that when participants thought that they could quit smoking, they might also think that their likelihood of experiencing severe COVID-19 symptoms would become lower and thus, less likely to take COVID-19 precautions.

Contrary to the EPPM, the perceived threat of COVID-19 negatively predicted intentions to take protective behaviors against COVID-19. One study found a similar negative association [46]. The authors explained that because COVID-19 is a novel public health emergency and people have limited knowledge about the disease, perceived threat of the disease may evoke higher irritation, disgust, and puzzlement, which may distract people from taking protective actions [46]. Moreover, So [47] theorized that when the perceived threat is high but the perceived efficacy is low, individuals’ dispositional coping style determines whether they will seek more information about the threat or reject the message. In a natural setting such as the context of COVID-19, people’s coping style may moderate how the perceived threat of the disease predicts behavioral intentions. Future studies should continue examining EPPM in a natural context for improvement.

### 4.3. Predicting Fear and Fatalism

Fear has been conceptualized as the key mechanism to motivate behavior in the EPPM [14]. The EPPM theorizes that fear directly predicts the fear control responses but is only indirectly related to danger control responses through feedback to perceived efficacy. Our findings pointed to the more constructive role of fear. Specifically, feelings of fear while looking at the message positively predicted quit intentions (danger control outcome) and were not related to fatalism (fear control outcome). First, the direct positive link between fear and danger control outcome has been documented in prior research [17], including in the context of COVID-19 [48]. The emotional response might have been overwhelming at the time of our research when mass and social media continuously reported increasing fatalities from the pandemic, making the severe risk so salient that it motivated participants to quit smoking to avert this threat.

It should be noted that our messages contained information and photos of patients with COVID-19, smoking hospitalizations, and deaths, which possibly conjured up an immense threat in participants’ minds. Fear of being the most at-risk group might spur participants’ quit intentions. In addition, the lack of a significant relationship between fear and fatalism also corresponds to some previous studies. For example, past research has found only mixed support for the proposition that fear directly predicts fear control responses (see Popova, 2012 for review [30]). As theorized previously [49], it is possible that those who engage in fear control response (e.g., fatalism) have already used this mechanism to reduce their fear; therefore, they do not feel as much fear while looking at the messages.

Our results showed a positive correlation between quit intentions (danger control outcome) and fatalism (fear control outcome). While the two types of outcomes have been theorized to be negatively correlated [15], prior research found that a small proportion of participants who smoke (around 3%) exhibited both adaptive and maladaptive responses in their written open-ended responses to fear appeal messages (cigarette warning labels) [49]. In addition, a longitudinal study of people who smoke in Australia, Canada, Mexico, and the United States, found that reactance and avoidance of warning labels on cigarettes (fear control responses) were associated with making a quit attempt (danger control response) [50]. While we did not have evidence to discuss mechanisms underpinning this positive association, we conjecture that participants might have been primed to consider fatalism in the context of COVID-19 and became so fatalistic about the disease that they wanted to quit smoking to gain a sense of control over their life. Future studies should continue this research direction to shed light on the association between danger control response and fear control response.

### 4.4. Implications for Messages

Using messages that combine information about two related health threats, such as COVID-19 and smoking, is a promising avenue to capitalize on the “teachable moment” of the pandemic and make individuals who smoke more aware of the harms that smoking is doing to their bodies, particularly in situations where there are other dangers in the environment. While this study focused on the perceptions of threat and efficacy rather than on threat and efficacy as message features [30], messages can be designed to influence perceived threat and efficacy. Given that perceived efficacy consistently had positive associations with desired danger control responses, messages should prioritize information on both smoking and COVID-19 efficacy, such as how quitting smoking can be protective against getting more severe COVID-19, resources available to make quitting easier, and how easy measures are effective at preventing becoming infected with COVID-19.

Whether messages emphasize the threat should be assessed carefully, taking the findings from our study in the context of other findings related to COVID-19, the changing situation with the pandemic, and the goals of the communication campaign. We found that COVID-19 threat positively predicted quit intentions. Moreover, fear evoked by perceived threat was positively related to quit intentions. Even fatalism (the “undesired” fear control response) was positively correlated with quit intentions. Thus, it seems that if the goal is to motivate smoking cessation, messages can emphasize the increased severity of COVID-19 for people who smoke. However, if the goal is to promote COVID-19-related protective behaviors, the threat might be potentially ineffective, likely due to the already high pre-existing levels of threat and fear [51], as was the case at the time of the study in August 2020.

### 4.5. Limitations

This study was conducted in August 2020, before COVID-19 vaccines became available. Thus, we did not measure intentions to vaccinate as a protective outcome. In addition, COVID-19 is a moving target. Later developments, such as the emergence of novel variants and the spread of misinformation, likely affect the variables in this study and should be examined in future research. Finally, the data are cross-sectional, which precludes making causal claims. The convenience nature of the sample also limits the generalizability of the study.

## 5. Conclusions

Combining multiple threats in a single message might be a promising strategy to motivate smoking cessation during the COVID-19 pandemic. Including information on effective and easy ways to address both threats seems to be the crucial mechanism through which these messages operate. In this context, fear evoked by the messages played a constructive role and was associated with greater smoking-cessation-related intentions.

## Figures and Tables

**Table 1:** Study variable descriptive statistics and bivariate correlations.

Variables	1	2	3	4	5	6	7	8
1. Smoking threat								
2. Smoking efficacy	0.33 ***							
3. COVID-19 threat	0.60 ***	0.27 ***						
4. COVID-19 efficacy	0.36 ***	0.46 ***	0.34 ***					
5. Fear	0.26 ***	0.26 ***	0.38 ***	0.19 ***				
6. Fatalism	0.20 ***	0.16 ***	0.26 ***	0.02	0.17 ***			
7. Quit intentions	0.27 ***	0.53 ***	0.32 ***	0.30 ***	0.35 ***	0.23 ***		
8. COVID intentions	0.22 ***	0.20 ***	0.18 ***	0.61 ***	0.15 ***	−0.06	0.16 ***	
Mean	6.34	5.9	5.74	7.34	5.56	4.09	5.19	3.59
*SD*	1.92	1.66	1.88	1.52	2.67	1.44	2.19	0.62
Range (*Min*–*Max*)	1–9	1–9	1–9	1–9	1–9	1–7	1–9	1–4

Note: *N* = 747. Smoking threat = perceived threat of smoking, smoking efficacy = perceived efficacy of quitting smoking, COVID-19 threat = perceived threat of COVID-19, COVID-19 efficacy = perceived efficacy of COVID-19-protective behaviors, COVID intentions = intentions to engage in COVID-protective behaviors. All items were measured on a 1 (*not at all*) to 9 (*extremely*) scale except for fatalism (1–7 scale) and COVID-19-protective intentions (1-4 scale). *** *p* < 0.001.

**Table 2:** Path analysis results.

Path	*B*	*SE*	β	*p*	95% CI	
					*LL*	*UL*
Smoking threat → Quit int	0.028	0.039	0.028	0.472	−0.048	0.105
COVID-19 threat → Quit int	0.104	0.038	0.104	0.007	0.029	0.180
Threat interaction → Quit int	0.013	0.027	0.015	0.636	−0.04	0.066
Smoking efficacy → Quit int	0.306	0.035	0.306	<0.001	0.237	0.376
COVID-19 efficacy → Quit int	0.101	0.039	0.101	0.009	0.025	0.177
Efficacy interaction → Quit int	0.070	0.027	0.094	0.008	0.018	0.122
Fear → Quit int ^^^	0.130	0.035	0.121	<0.001	0.062	0.199
Smoking threat → COVID int	0.011	0.038	0.011	0.779	−0.064	0.086
COVID-19 threat → COVID int	−0.090	0.037	−0.092	0.015	−0.163	−0.017
Threat interaction → COVID int	−0.051	0.026	−0.061	0.053	−0.103	0.001
Smoking efficacy → COVID int	−0.064	0.034	−0.065	0.062	−0.131	0.003
COVID-19 efficacy → COVID int	0.502	0.038	0.512	<0.001	0.428	0.577
Efficacy interaction → COVID int	−0.055	0.026	−0.075	0.035	−0.105	−0.004
Smoking threat → Fear	0.033	0.041	0.035	0.420	−0.047	0.113
COVID-19 threat → Fear	0.150	0.040	0.160	<0.001	0.071	0.228
Threat interaction → Fear	0.024	0.028	0.031	0.391	−0.031	0.079
Smoking efficacy → Fear	0.126	0.037	0.135	0.001	0.054	0.198
COVID-19 efficacy → Fear	−0.033	0.041	−0.035	0.417	−0.112	0.047
Efficacy interaction → Fear	0.010	0.028	0.014	0.727	−0.045	0.064
Fear → Fatalism	0.029	0.041	0.027	0.477	−0.051	0.109
Smoking threat → Fatalism ^^^	0.108	0.045	0.106	0.018	0.019	0.197
COVID-19 threat → Fatalism ^^^	0.122	0.045	0.123	0.007	0.034	0.21
Threat interaction → Fatalism ^^^	0.112	0.031	0.134	<0.001	0.051	0.174
Smoking efficacy → Fatalism ^^^	0.084	0.041	0.085	0.042	0.003	0.165
COVID-19 efficacy → Fatalism ^^^	−0.148	0.045	−0.150	0.001	−0.236	−0.059
Efficacy interaction → Fatalism ^^^	−0.126	0.031	−0.171	<0.001	−0.187	−0.065

Note: *N* = 747. Smoking threat = perceived threat of smoking, COVID-19 threat = perceived threat of COVID-19, threat interaction = the interaction between perceived threat of smoking and COVID-19, smoking efficacy = perceived efficacy of quitting smoking, COVID-19 efficacy = perceived efficacy of COVID-19-protective behaviors, efficacy interaction = the interaction between perceived efficacy of quitting smoking and COVID-19-protective behaviors, quit int = intentions to quit smoking, COVID int = intentions to engage in COVID-19-protective behaviors. ^^^ indicates paths that were added to the original model based on modification indices.

## Data Availability

Data are available at https://scholarworks.gsu.edu/sph_datasets/2/ (accessed on 14 January 2023).
